# An electro-responsive hydrogel for intravascular applications: an in vitro and in vivo evaluation

**DOI:** 10.1007/s10856-015-5598-9

**Published:** 2015-10-16

**Authors:** Peter Verbrugghe, Jelle Verhoeven, Walter Coudyzer, Eric Verbeken, Peter Dubruel, Eduardo Mendes, Frank Stam, Bart Meuris, Paul Herijgers

**Affiliations:** Department of Cardiac Surgery, UZ Leuven, Herestraat 49, 3000 Louvain, Belgium; Department of Radiology, UZ Leuven, Louvain, Belgium; Department of Pathology, UZ Leuven, Louvain, Belgium; Chemistry Department, Ghent University, Ghent, Belgium; Chemical Engineering Department, Delft University, Delft, The Netherlands; Tyndall National Institute, University College Cork, Cork, Ireland

## Abstract

There is a growing interest in using hydrogels for biomedical applications, because of more favourable characteristics. Some of these hydrogels can be activated by using particular stimuli, for example electrical fields. These stimuli can change the hydrogel shape in a predefined way. It could make them capable of adaptation to patient-specific anatomy even post-implantation. This is the first paper aiming to describe in vivo studies of an electro-responsive, Pluronic F127 based hydrogel, for intravascular applications. Pluronic methacrylic acid hydrogel (PF127/MANa) was in vitro tested for its haemolytic and cytotoxic effects. Minimal invasive implantation in the carotid artery of sheep was used to evaluate its medium-term biological effects, through biochemical, macroscopic, radiographic, and microscopic evaluation. Indirect and direct testing of the material gave no indication of the haemolytic effects of the material. Determination of fibroblast viability after 24 h of incubation in an extract of the hydrogel showed no cytotoxic effects. Occlusion was obtained within 1 h following in vivo implantation. Evaluation at time of autopsy showed a persistent occlusion with no systemic effects, no signs of embolization and mild effects on the arterial wall. An important proof-of-concept was obtained showing biocompatibility and effectiveness of a pluronic based electro-responsive hydrogel for obtaining an arterial occlusion with limited biological impact. So the selected pluronic-methacrylic acid based hydrogel can be used as an endovascular occlusion device. More importantly it is the first step in further development of electro-active hydrogels for a broad range of intra-vascular applications (e.g. system to prevent endoleakage in aortic aneurysm treatment, intra-vascular drug delivery).

## Introduction

In recent years there have been significant advances in the development of hydrogels for biomedical applications. These water-swollen macromolecules have many advantages. First of all they can swell/deswell several times their original volume. They are stable in aqueous and biofluids at physiological temperature, pH, and ionic strength [1]. Due to their high water contents and soft consistency hydrogels resemble natural living tissue more than any other class of synthetic biomaterials and several of these gels are found to be biocompatible [2–4]. Moreover, their physical and chemical properties can vary with composition [5, 6], and they can conform to various shapes [7]. Other novel functionalities of these polymers could also possibly be utilised. For example the shape-memory effect, these polymers change their shape in a predefined way when exposed to a suitable stimulus. This stimulus can be a chemical reaction, a temperature change, electrical fields, solvents and light [8, 9]. The most commonly studied biomedical application for this type of hydrogel is drug delivery, where an applied stimulus causes the hydrogel to deswell which releases a specified volume of drug [10]. A potential new application could be creating an intravascular occlusion by using the hydrogel’s ability to expand or shrink when stimulated. In fact several medical procedures require the occlusion of a blood vessel: treatment of vascular bleeding, aneurysm sac occlusion, etc. Previously non-stimuli responsive hydrogels have been used to coat a coil [11] or to be injected in an aneurysm sac [12]. This paper evaluates the biocompatibility and vascular occlusion capability of a pluronic-methacrylic acid based hydrogel that exhibits strong electro-mechanical actuation [13].

## Materials and methods

### Hydrogel

The synthesis of the hydrogel (PF127/MANa) used in this paper has been described in detail [13]. Pluronic-bismethacrylate (PF127-BMA) powder 15 wt% was mixed with methacrylic acid sodium salt (MANa) 10 wt% in nitrogen gassed de-ionised water and cooled for 8 h. Free radical polymerization was obtained by adding an initiator, 1 M ammonium persulfate (APS) (Sigma Aldrich A3678). As an accelerator 1 M tetramethylethylenediamine (TEMED) (Sigma Aldrich T9281) solution was used. The mixture was then placed in a PVC mould and refrigerated for 1 h. Finally, the created hydrogel was cured at 37 °C for 3 h. After polymerization the hydrogel was removed from the mould and transferred to a phosphate buffered saline solution (PBS). This solution was refreshed three times a day for three consecutive days. Thereafter the hydrogel was air dried for three consecutive days.

### Biocompatibility

#### Haemolytic tests

##### Haemoglobin determination

To determine the free haemoglobin concentration the Drabkins reagent method was used in accordance with the international standard (ASTM F756-08) [14]. Drabkins reagent converts haemoglobin to the cyanoderivative [15]. The colour intensity measured at 540 nm is proportional to the total haemoglobin concentration. Fresh human blood, anticoagulated with acid citrate dextrose, from three donors, was mixed and used. The free plasma haemoglobin concentration was lower than 2 mg/ml (0.53 mg/ml). The blood was diluted with an appropriate amount of calcium- and magnesium-free PBS to adjust the total haemoglobin content to 10 ± 1 mg/ml. For the direct test and the extract test a haemolytic index was calculated using the following equation:

$$\% Hemolysis = \frac{(Hb\;testsample - Hb\;negativecontrol)}{(Hb\;positive\;controm - Hb\;negative\;control)} \times 100$$

##### Extract testing

A dried cylindrical hydrogel sample (Ø3.5 mm × 14.3 mm) was placed in a tube with 20 ml PBS for 24 h at 37 °C. Then 0.1 ml of diluted blood added to 0.7 ml of extract was added to the tube, and this mixture was placed in a test tube rack for 3 h at 37 °C. The tube was gently inverted three times every 30 min to maintain good contact with the blood and extract. At the end of the incubation time the sample was centrifuged for 15 min at 750 G. The supernatant was carefully removed and 0.250 ml was added to 0.250 ml Drabkins solution. This mixture was allowed to stand for 15 min at 37 °C. After this 0.1 ml of this solution was placed in a 96-well plate and the absorbance was recorded with a spectrophotometer at a wavelength of 540 nm. The haemoglobin concentration of the supernatant was calculated using a standard curve. Six test samples, 6 positive controls (H_2_0), and 6 negative controls (extract of high density polyethylene (HDPE)) were evaluated.

##### Direct testing

Cylindrical hydrogel samples, with dimensions Ø3.0 mm × 7.6 mm, were inserted in tubes immersed in a mixture of 0.7 ml PBS and 0.1 ml of diluted blood for 3 h at 37 °C. Each tube was gently inverted three times every 30 min to maintain good contact between blood and material. At the end of the incubation time the samples were centrifuged for 15 min at 750 G. The remaining steps were identical to the steps described for the “Extract testing”.

#### Cytotoxicity

##### Culture conditions

Mouse L929 fibroblasts were obtained from European Collection of Cell Cultures (ECACC No. 88102702) and routinely cultured in a minimal essential medium supplemented with 10 % foetal calf serum, 1 % MEAA, 1 % penicillin G, 1 % streptomycin sulphate, 10 μl/ml l-glutamine. Cultures were maintained in an atmosphere of 5 % CO_2_ in air at 37 °C, and the medium was changed every 48 h.

##### Extract testing

The in vitro cytotoxicity was evaluated by means of the dilution test method, ISO 10993-5 [16]. This test exposes fibroblasts grown to near-confluence to fluid-extracts from the material under investigation. Cells seeded in a concentration of 1.10^5^/ml were cultured for 24 h in a 96-well microplate. Thereafter the medium was exchanged with the extract of the test samples. These extracts were obtained by placing a cylindrical gel sample (Ø7.6 mm × 25 mm), swollen in PBS, into 20 ml of culture medium for 24 h at 37 °C. Dilutions (1/1, 1/2, 1/4, 1/10, 1/100, 1/1000) were fabricated with sterilized culture medium. As a negative control, a culture medium having been in contact with HDPE was used (50 × 50 × 1 mm sample in 17.3 ml medium). As a positive control phenol (6.4 g/L) was used. For each condition six samples were tested. After 24 h of incubation, cell viability was assessed with the quantitative colorimetric MTT assay (Vybrant MTT Cell Proliferation Assay Kit, Molecular Probes) [17, 18]. The cell viability percentage was reported as function of control.

#### In vivo evaluation

##### Implantation

**Fig. 1:**
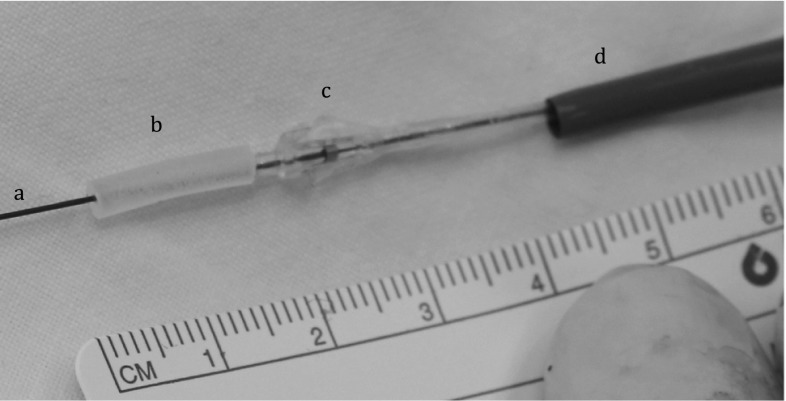
Delivery device with guidewire (**a**), hydrogel (**b**), occlusion balloon (**c**), and delivery sheath (**d**)

The Ethical Committee for animal experiments at the KU Leuven approved the experimentation (number P144-2010). Hydrogel cylinders (Ø3 mm × 15 mm) were implanted in the carotid artery of three sheep in a minimal invasive way. The sheep were sedated with intramuscular injection of ketamine (15 mg/kg). Anaesthesia was induced with isoflurane 5 %, the animal was intubated and an oral-gastric tube was placed. Anaesthesia was maintained with isoflurane (2–4 %). An intravenous line and arterial line in both ears were inserted. Heart rate, blood pressure, end-tidal CO_2_, and blood O_2_ saturation were constantly monitored. The left side of the neck was shaved. The carotid artery was isolated proximal and distal from the intended implant site of the hydrogel. Flow measurements were performed using a Doppler-flow probe. Heparine (100 U/kg) was administered before sheath placement. Proximal a 4 Fr sheath was placed for pressure measurement and angiography. Distally an 11 Fr sheath was placed for hydrogel delivery and pressure measurements. The delivery system, as shown in Fig. 1, consisted of a 9 Fr conventional sheath, straight 0.014-inch guidewire, and an occlusion balloon. After reaching the target destination, the occlusion balloon was inflated. Angiography was used every ten minutes to assess the occlusion status. After obtaining occlusion all attributes used for the implantation procedure were removed and a final angiography, pressure-, and flow measurement were performed. The sheaths were retracted and the puncture side of the carotid artery was closed with stiches. The incisions were closed in layers.

##### Clinical and macroscopic evaluation

The health status of the animals was checked on a daily basis. Blood samples were taken before implantation, and 2 and 4 weeks after implantation. The blood samples were analysed for blood cell count, ion content, signs of haemolysis (free haemoglobin, LDH), and blood urea nitrogen. Four weeks after implantation the sheep were sedated with intramuscular injection of ketamine and anaesthesia was performed using isoflurane (2–4 %). Computer tomography was conducted and an experienced radiologist performed an evaluation on the carotid arteries, and brains looking for signs of embolization. Next the entire carotid arteries were isolated. Proximal and distal from the hydrogel an arterial catheter was placed for pressure measurement. Flow was measured using a Doppler flow device. After this, heparin was administered and the animal was euthanized using a high-dose pentobarbital and potassium. The left carotid artery and surrounding tissue were subsequently explanted. A full autopsy was performed evaluating all organs to detect any signs of gel embolization or toxic effects.

##### Microscopic evaluation

The explanted hydrogel and vessel were evaluated macroscopically, separated and processed for histological analysis by haematoxylin and eosin staining. An experienced pathologist performed an evaluation of the architecture and inflammatory response of the explanted carotid artery. Samples that had been in direct contact with the hydrogel were compared with samples from unaffected carotid artery sections (taken 5 cm more proximal). An evaluation of the architecture and reaction of the intima; inflammation, fiber architecture and thickness of the media; and inflammatory reaction within the adventitia was carried out. A scoring system from 0 to 3 was used to describe the inflammatory response and fiber disruption. “0” indicating no inflammation or intact fiber architecture, and “3” a pronounced inflammation or disruption of fibres. The observer was blinded for the sample group.

## Results

### Biocompatibility

#### Haemolytic tests

Extract and direct testing of the hydrogel gave no indication that the material has haemolytic properties. The amount of free haemoglobin, after contact with an extract of the hydrogel or with the hydrogel surface was comparable with the negative control. This was represented in the percentage of haemolysis: 0.06, and 1.86 %, respectively.

#### Cell toxicity tests

**Fig. 2:**
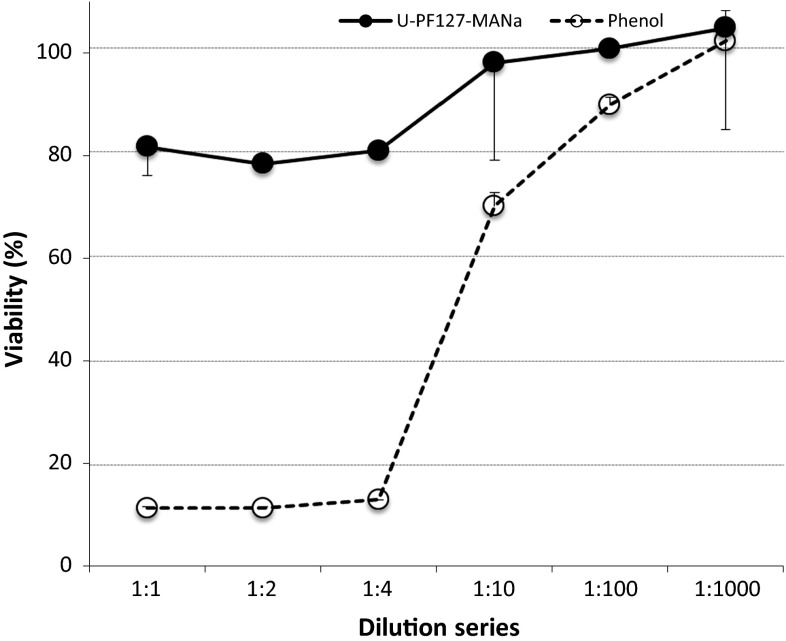
Viability, with standard deviation, of a cell culture after contact with a diluted extract of the hydrogel, versus a positive control (phenol)

Figure 2 shows fibroblast viability after 24 h of incubation in an extract of the hydrogel. The viability of the cells after contact with the extract is 81 per cent. The positive control, phenol, is toxic until a dilution of 1 over 10 is reached.

## In vivo evaluation

### Clinical and macroscopic evaluation

**Fig. 3:**
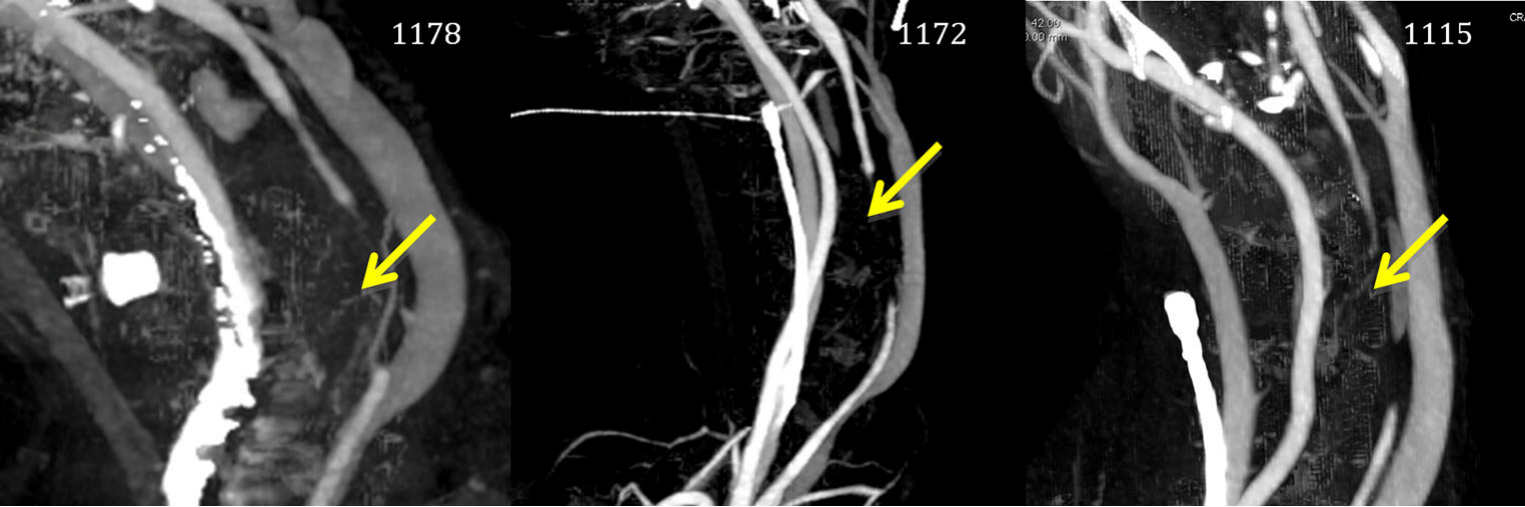
CT images of the occluded carotid artery

**Fig. 4:**
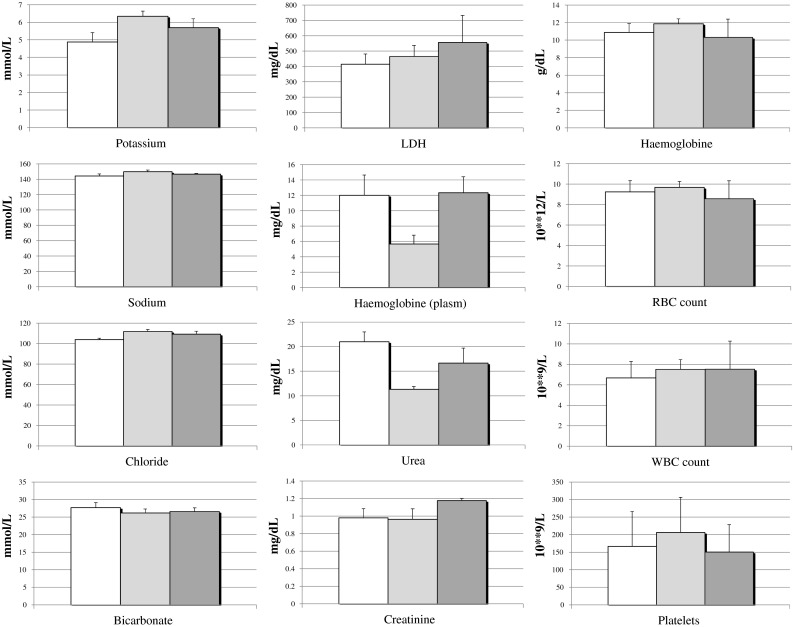
Average blood values, with standard deviation, 0 days (*white*), 14 days (*grey*), and 28 days (*dark grey*) after implantation

The average flow in the carotid artery before occlusion was 248 ± 4 ml/min. One hour after hydrogel implantation, flow was zero in all three sheep. Perioperative angiography confirmed this finding. Doppler flow measurements and angiography (Fig. 3) 4 weeks later showed a persistent occlusion. At that time some filling defect and decrease in vessel diameter was visible proximal to the hydrogel. Evaluation of the pressure directly after implantation showed a slightly lower pressure distal from the hydrogel (mean pressure: 65 ± 16 mmHg) compared to the proximal pressure (mean pressure: 74 ± 21 mmHg). Four weeks after implantation the difference in mean arterial pressure in front and behind the hydrogel is still measurable, 74 ± 17 and 62 ± 14 mmHg, respectively. Daily evaluation showed no evidence of any health problems in any off the sheep. There were no signs of organ failure, stroke or infection. Blood sample evaluation (Fig. 4) gave normal values throughout the experiment. Ion concentrations were normal. There were no indications of haemolysis, as lactate dehydrogenase (LDH) and plasma haemoglobin levels were normal. No indication of kidney failure either as urea and creatinin were low. There was also no evidence of cholestatic liver disease; bilirubin levels were below 0.18 mg/dL. All blood cell counts were normal, without any sign of inflammation and the number of white blood cells (WBC) being low. Furthermore, cerebral evaluation by an experienced radiologist did not show any sign of cerebral infarction caused by embolization.

**Fig. 5:**
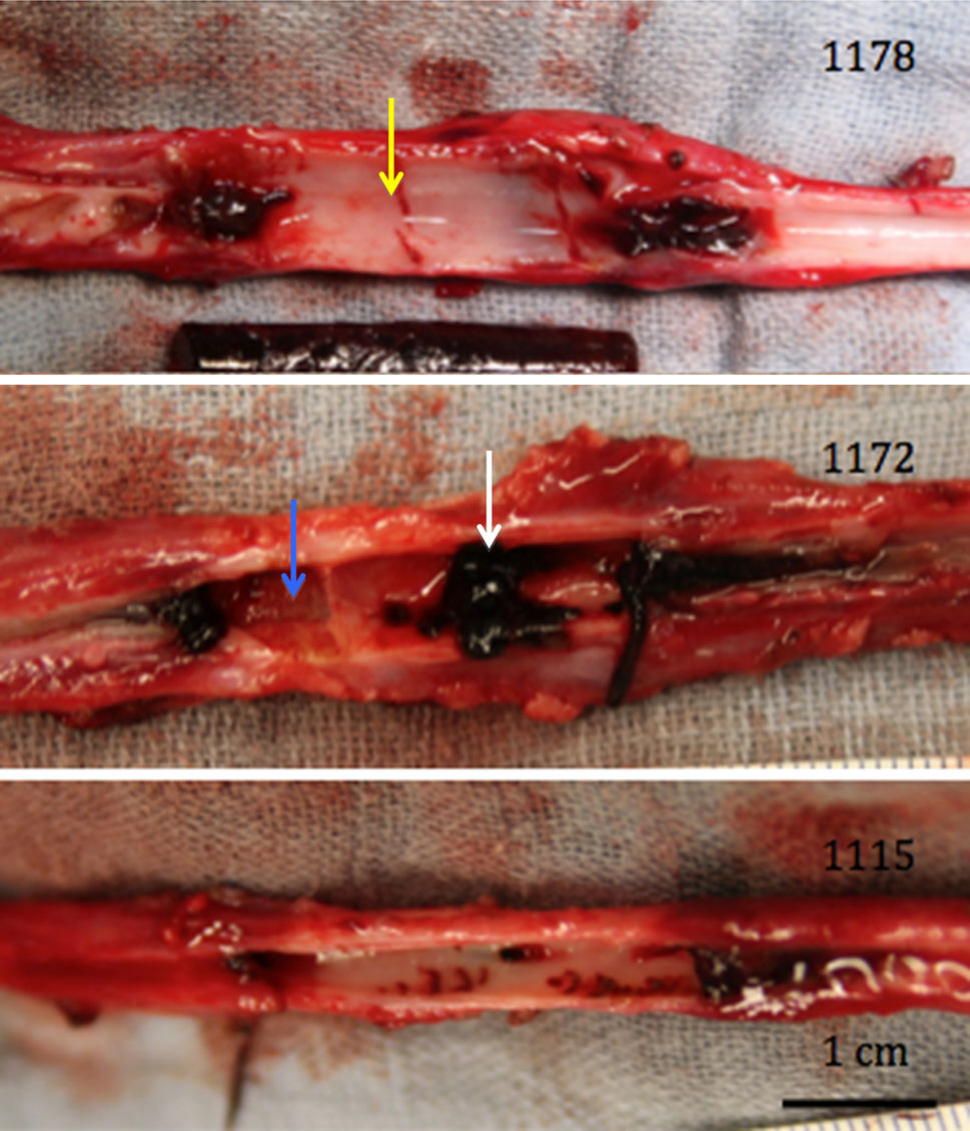
Photographs of the explanted carotid arteries. Thrombi can be seen proximal and distal from the place where the hydrogel has been. In sheep 1178 there were two vertical cracks (*yellow arrow*), in sheep 1172 there were thrombi at the vessel wall (*white arrow*) and an ulcer (*blue arrow*) (Color figure online)

Macroscopic evaluation, at time of explantation, showed an intact carotid artery. There were no firm adhesions of the vessel to its surrounding. Mild adhesions were seen a distance away from the hydrogels at the dissection and sheath insertion locations. The hydrogels were not attached to the vessel wall and fell out when the vessel was opened. There were small thrombi adjacent to both sides of the hydrogel (Fig. 5). Around the hydrogel there was a remarkable thrombus in one of the sheep (ID: 1172). Macroscopic evaluation of the vascular wall showed impairments in two of the sheep. In sheep 1178 there were two radial cracks, and in sheep 1172 there was a deep ulcer at the distal side of the hydrogel. Full autopsy could not reveal any sign of embolization, infarction, nor bleeding in the organs.

### Microscopic evaluation

**Fig. 6:**
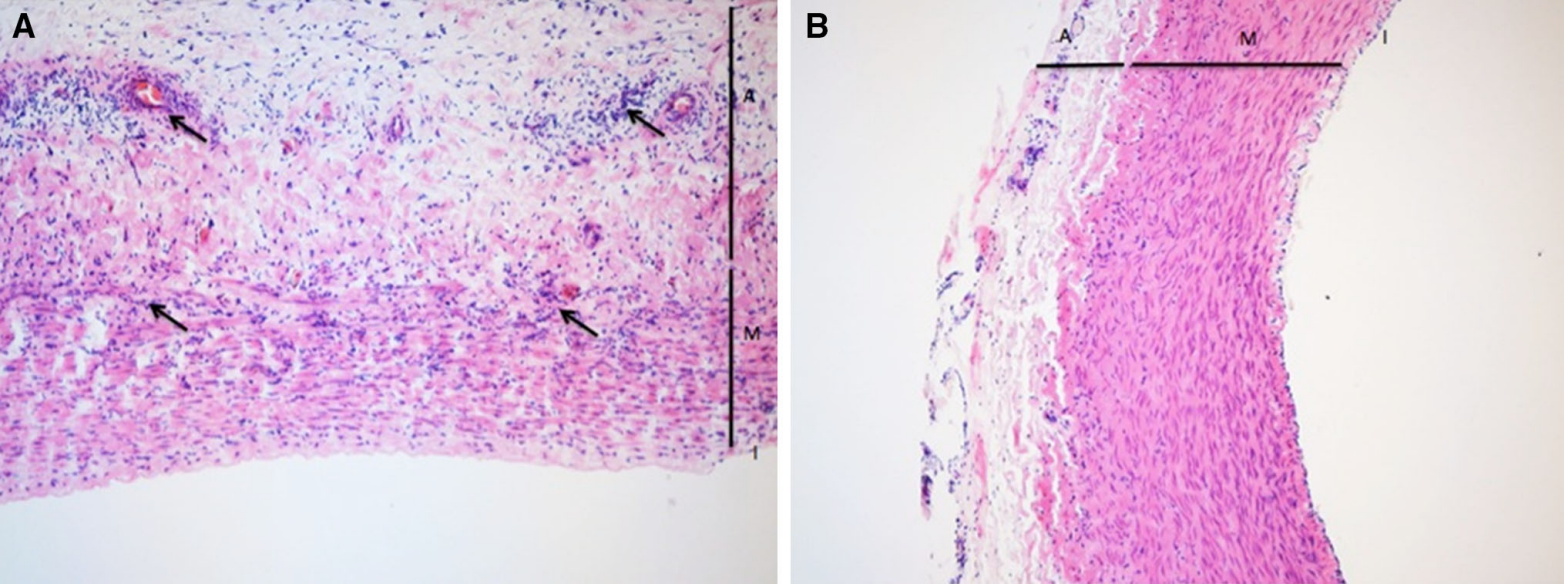
**a** Gel sample showing a cellular reaction in media and adventitia (*arrows*); **b** Control sample of a normal carotid artery (I: intima; M: media; A: adventitia; H&E staining, ×100)

An overview of the microscopic evaluation is depicted in Table 1. Microscopy of the vessels showed that the intima was morphologically intact in all randomly taken samples. There was a mild inflammatory reaction in the media of samples that were in contact with the hydrogel (Fig. 6). No fibrotic reaction could be visualized and tissue fibres were intact. A mild inflammatory reaction was also visualized in the adventitia in comparison with the unaffected carotid artery. A specific evaluation of the ulcer in sheep 1172 showed disruption of the intima and an extensive fibrotic and inflammatory reaction in the media. There was a moderate decrease in media thickness after contact with the hydrogel with a mean value of 268 μm ± 131, versus 545 μm ± 102 in the control samples, respectively.

**Table 1:** Overview of the pathology analysis of the carotid vessel wall

	Intima	Media			Adventitia
	Reaction	Inflammation (0–3)	Fibres (0–3)	Thickness (μm)	Inflammation (0–3)
Gel contact	0.33	1.67	0.00	307 ± 129	1.67
Normal	0.00	0.00	0.00	593 ± 298	0.00

## Discussion

Hydrogels for medical applications represent a substantially under-investigated field of research. But there is an increasing interest as new hydrogel functionalities are becoming available that might open up new medical device opportunities. Some of these hydrogel features (e.g. soft consistency, biostability) are ideal for the intravascular use of these hydrogels. Previously already some non-stimuli responsive hydrogels have been used [11, 12], but the more novel functionalities of today may broaden the field of intravascular applications.

Per definition a biomaterial is a polymer whose surface is in direct contact with the biological system, so the biocompatibility of this material should be assessed [19]. The initial building block that was selected for developing a suitable electro-responsive hydrogel was F127 (i.e. a specific type of pluronic), a material known to be “biocompatible” and approved by the FDA (Food and Drug Administration). The pluronic material was bound with bismethacrylate (PF127-BMA) and polymerized with methacrylic acid, using the APS/TEMED initiating system, to obtain the electro-responsive behaviour [13]. A possible toxic residue is bismethacrylate, as this remains in the gel if there is incomplete polymerization. Another possible cause of toxicity could be the redox initiating system [20]. To decrease the toxicity potential a rinsing procedure (PBS solution refreshed three times a day for three consecutive days) was introduced. Evaluation of haemolytic and cytotoxic behaviour of the PF127/MANa was carried out according to international guidelines [14, 16]. The percentage of haemolysis was less than 2 % for the direct and indirect tests. Therefore PF127/MANa is considered non-haemolytic. Both direct and extract testing were performed as both serve a different goal. The direct testing focuses on the haemolytic features of the hydrogel as a whole, including the materials surface properties which are important in the interaction with a biological environment and artificial materials [21]. The indirect or extract testing is important to define haemolytic agents, released within the environment of the gel after implantation. There was no indication of toxicity of the hydrogel as there was no reduction of cell viability by more than 30 %, which is considered as cut-off point for cytotoxic effects. In vitro experiments did not prove full biocompatibility as more complex interactions with foreign material dipped in blood or tissue fluids occur. It leads to adsorption of biomolecules, cells and usually proteins on its surface within even a few seconds of immersion, followed by secondary interactions like a cellular response and thrombus formation [22]. For this reason in vivo experiments were performed.

By endovascular implantation of the PF127/MANa material in the carotid artery of sheep, it was shown that an occlusion could be obtained within 1 h after implantation. Four weeks after implantation Doppler and angiographic evaluation proved complete occlusion. There was a mild pressure drop over the hydrogel implant when occlusion occurred. The reason that pressure distal from the occlusion is relatively high is due to a well-developed collateral cerebral circulation in sheep. At time of explantation there were no signs of disintegration or fragmentation of the hydrogel. Moreover, cerebral imaging and investigation of organs at time of autopsy could not show any evidence of embolization. There was a limited amount of thrombi adjacent to the hydrogel device. This was probably caused by no-flow conditions in the carotid artery due to an effective occlusion. The presence of hydrogel in the blood may cause loss of, or damage to, red blood cells and may produce increased levels of free plasma haemoglobin capable of inducing toxic effect or other effects which may stress the kidneys or other organs. Frequent biochemical analysis after implantation did not show any haemolytic or other undesired systemic effects from the hydrogel. A macroscopic evaluation of the medium-term effects of the hydrogel on the vascular wall was carried out. The PF127/MANa gel did not show to adhere to the vessel wall and caused no macroscopic reactions outside the vessel. The radial cracks at the inside of the vessel might have been caused by traction during explantation of the artery. It is believed that, because of the already extensive fibrotic reaction, the defect in the intima of sheep 1172 is a remnant of damage caused by the sheath or guidewire at time of implantation. Microscopy showed that the hydrogel provoked a mild inflammatory reaction in the media and adventitia of the artery. An intact morphology of the vessel wall was found, but with a mild decrease in media thickness.

To conclude, this study showed that this electro-responsive hydrogel, which can expand and contract by several times its original volume, can cause an effective arterial occlusion whereby the hydrogel is relatively inert to the vessel wall. As predefined geometrical changes can be obtained by application of a small voltage to the hydrogel, it is believed that this material may add significant benefits over existing occlusion devices. Moreover, its geometric adaptation capacity might be of use for other minimal invasive vascular applications. One example is prevention of endoleakage after endovascular treatment of abdominal aortic aneurysms [23]. A second potential application is using the material for intravascular drug delivery [24, 25].
